# Effects of Sediment Chemical Properties on Phosphorus Release Rates in the Sediment-Water Interface of the Steppe Wetlands

**DOI:** 10.3390/ijerph14111430

**Published:** 2017-11-22

**Authors:** Jing He, Derong Su, Shihai Lv, Zhaoyan Diao, Jingjie Xie, Yan Luo

**Affiliations:** 1 Grassland Resources and Ecology Research Center, Beijing Forestry University, Beijing 100083, China; hejing_606@163.com (J.H.); xiejingjie@163.com (J.X.); luoyan1101@163.com (Y.L.); 2 State Environmental Protection Key Laboratory of Regional Eco-Process and Function Assessment, Chinese Research Academy of Environmental Sciences, Beijing 100012, China; lvsh1963@163.com (S.L.); diaozhy@126.com (Z.D.)

**Keywords:** steppe wetland, sediment chemical properties, phosphorus release rates, sediment–water interface, microbial biomass

## Abstract

Rising temperature causes a process of phosphorus release, which can be characterized well using phosphorus release rates (V_P_). The objective of the present study was to investigate the major factors affecting sediment phosphorus release rates through a wetland habitat simulation experiment. The results showed that the V_P_ of different wetland sediments were different and changed with the order of W–R (river wetland) > W–L (lake wetland) > W–M (grassy marsh wetland) > W–A (reservoir wetland). The main driving factors which influenced sediment phosphorus flux velocity in the sediment–water interface were sediment B-SO_4_^2−^, B-MBN and A-MBP content. Path analysis and determination coefficient analysis indicated the standard multiple regression equation for sediment phosphorus release rates in the sediment–water interface, and each main factor was Y = −0.105 + 0.096X_1_ + 0.275X_2_ − 0.010X_3_ (*r* = 0.416, *p* < 0.01, *n* = 144), where Y is sediment phosphorus release rates; X_1_ is sediment B-SO_4_^2−^ content; X_2_ is sediment B-MBN; and X_3_ is sediment A-MBP content. Sediment B-SO_4_^2−^, B-MBN and A-MBP content and the interaction between them were the main factors affecting sediment phosphorus release rates in the sediment–water interface. Therefore, these results suggest that soil chemical properties and microbial activities likely play an important role in phosphorus release rates in the sediment–water interface. We hope to provide effective scientific management and control methods for relevant environmental protection departments.

## 1. Introduction

Eutrophication, caused by nutrient-rich inputs through both point-source discharges and non-point loading, threatens most freshwater bodies [1,2,3,4]. Generally, when the ratio of nutrition (N)/phosphorus (P) in a lake is larger than 10, phosphorus is the limited element of eutrophication [5]. However, the decline of P content in freshwater will cause the decline of the dominant position of cyanobacterial material [6,7,8,9,10]. The source of phosphorus in a freshwater body includes an internal source and an external source, and the internal source of phosphorus in a freshwater body has been proven to be a large proportion of the total phosphorus input [11,12,13,14].

Environmental factors e.g., temperature [15], pH [16], redox potential [17], Eh [18], P concentration gradient [19] and hydrological conditions [20] in the sediment–water interface would affect P release processes and release rates [21]. However, the results from different sediments are sometimes not the same because P release processes and release rates are significantly affected by both the physical and chemical properties of the sediments [22,23,24,25,26].

The Dauria steppe wetlands lie in the northern part of Central Asia and are ecologically strongly dependent on climate change. Most of the Dauria steppe area is located in north-east China and eastern Mongolia; the Russian part is referred to Zabaikalsky Province and the Buryat Republic [27]. The Dauria steppe wetlands is included in the Global 200 Ecoregions of the World as the *Dauria Steppe* with nine main wetland ecosystem services and key cultural value. The Hulunbeier steppe, which possesses large floodplain wetlands with reedbeds known as a breeding area for significant numbers of waterfowl and migratory birds, is an important area of the Dauria steppe wetlands in China [27]. The highest temperature of the Hulunbeier steppe occurs in July and is characterized by the synchronization of high temperature and ample precipitation [28]. Furthermore, an increase in temperature can enhance the internal phosphorus cycle in the sediments of wetlands, leading to a more serious deterioration of water quality [29,30,31]. Although it is well known that increased temperatures result in greater P release [32,33], little is known about the effect of physico-chemical properties on P release rates in the Hulunbeier Steppe wetland.

Therefore, the objective of the present experiment was to analyze the driving factors affecting sediment phosphorus release rates. It was also to investigate the relationship among these main factors which drive the sediment phosphorus release rates. It hopes to provide basic support for reducing and controlling phosphorus release from sediment in steppe wetland.

## 2. Materials and Methods

### 2.1. Study Site

**Figure 1:**
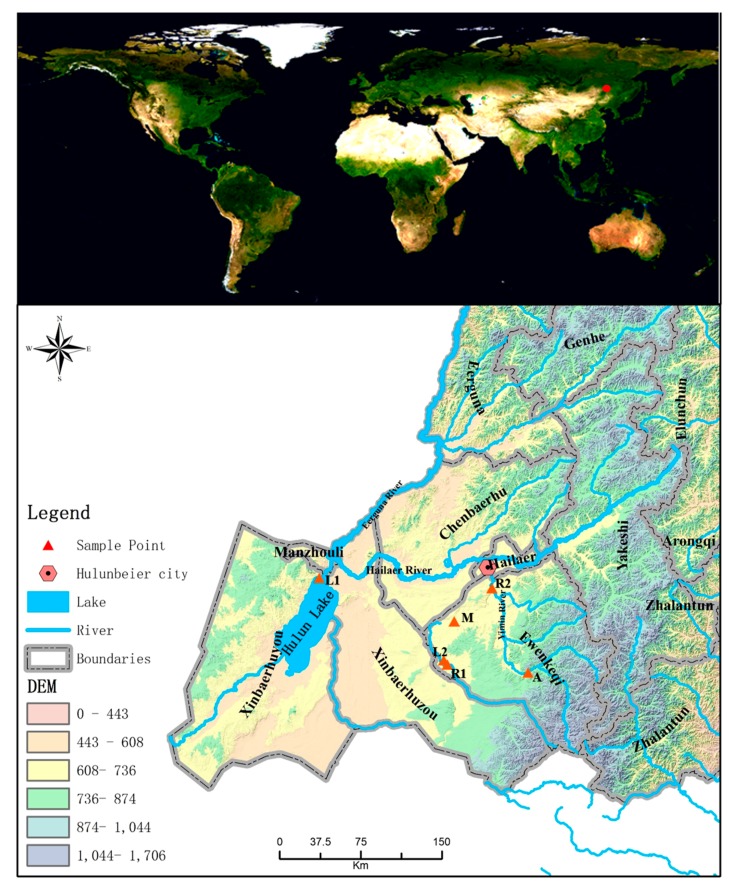
Location of the steppe wetland and distribution of sampling points.

This study was performed in the Hulunbeier steppe, which is located in inner Mongolia, north-eastern China (47°05′~53°20′ N, 115°31′~23°00′ E; 500–900 m above sea level) (Figure 1). The river–wetland, lake–wetland and marsh–wetland are the main wetland types in this region. The climate of this site is classified as continental: winters are cold and snowy and summers are warm and humid. The mean annual precipitation of the study site is 240–400 mm, and the mean annual temperature is −1–0 °C. Extreme air temperatures in winter can reach −25 °C, while in summer, temperatures can exceed 30 °C.

In middle July 2015 and July 2016, samples of sediments at 0~10 cm depth from 6 wetlands were collected with the ZYQ-WN wetland sediment sampler (Beijing GRASP Science and Technology Development Co., Beijing, China) in the Hulunbeier steppe. According to the classification system of the wetland convention, the sediments belonged to four types of wetlands (Figure 1 and Table 1): river wetland (R1 and R2), freshwater lake wetland (L1 and L2), grassy marsh wetland (M) and reservoir wetland (A). The paired overlying water was collected by the water sampler (JC-800, Juchuang, Qingdao, China). Sediment samples and overlying water samples were saved in plastic bags and taken within 3 h back to the laboratory for simulation experiments and the determination of their chemical properties.

### 2.2. Experimental Design

**Figure 2:**
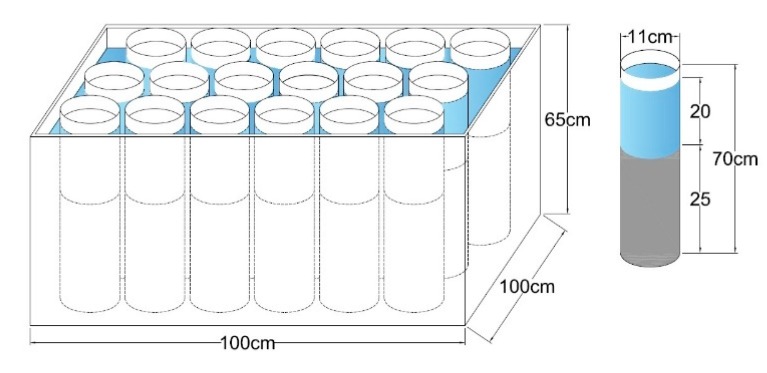
The design of the experimental wetland habitat simulation device.

The wetland habitat simulation device is mainly composed of an incubator box and polyvinyl chloride (PVC) wetland simulation pipes. The length, width and height of the box were 100, 100 and 65 cm; the PVC pipe specification was φ110 mm × 70 cm. The box was filled with water to 45 cm in depth which was lower than the PVC pipes (Figure 2). PVC wetland pipes were prepared before sampling. Each wetland pipe was designed to be filled with 25 cm of fresh sediment and 20 cm of the corresponding overlying water. All the PVC pipes were arranged and placed in the box for cultivation. The field sampling was conducted in July 2015 and July 2016; the average temperature and humidity during the culture period were 19.97 °C and 54.49%. Eight repetitions were designed for each wetland, and the total number of PVC pipes was 48.

Water samples of 100 mL were collected from each of the PVC pipes in 15 August 2015 and 12 August 2016, 30 days after the simulated experiment began, and saved in black bottles. Sediment samples of 300 g were collected from each PVC wetland pipes after the overlying water was sampled, and saved in plastic bags. All water and sediment samples were taken back to the laboratory within 3 h and frozen at −15 °C for the determination of their physical and chemical properties in the future.

### 2.3. Analysis Method of Water and Sediments

The total phosphorus (TP) content of the water was estimated by Molybdenum antimony spectrophotometric method. Also, the total phosphorus content of the sediment was estimated by the HClO_4_-H_2_SO_4_ Digestion–Antimony molybdenum spectrophotometry method. Inorganic phosphors (IP) were fractionated according to a conventional fractionation method, and IP was to be divided into 4 parts, including Al-P, Fe-P, Ca-P and Oc-P. The sediment samples were sieved (<2 mm), air-dried and mixed thoroughly. The sequential extraction procedure was started by extracting 1 g with 1 N NH_4_CL to remove the water soluble phosphorus. The sediment residue was further extracted with 0.5 N NH_4_F for determining AL-P and then with 0.1 N NaOH for determining Fe-P, with 0.5 N H_2_SO_4_ for determining Ca-P. Finally, the sediment residue was extracted with concentrated 0.3 N Na_3_C_6_H_5_O_7_·2H_2_O and 1 N NaHCO_3_ for determining Oc-P.

The microbial biomass C (MBC) and microbial biomass N (MBN) of the sediment were estimated by the fumigation–extraction method [34,35]. Fumigated and non-fumigated portions of 10 g moist sediment were extracted for 30 min by oscillator shaking at 200 revs min^−1^ with 40 mL 0.5 M K_2_SO_4,_ and filtered (Φ12.5 cm, Nourishment Technology Co., Ltd., Zhengzhou, China). Organic C and total N in the sediment extracts were measured after combustion by the potassium dichromate volumetric and thermal dilution method and the Kjeldahl nitrogen determination method, respectively. Sediment microbial biomass P with Olsen (0.5 M NaHCO_3_, pH 8.5) was also measured by the fumigation–extraction method [36] as described by Joergensen et al. [37].

(1) $$MBC=\frac{E_{C}}{k_{EC}}=\frac{F_{C}-N_{C}}{k_{EC}}$$

where *F_C_* = organic *C* extracted from fumigated sediments; *N_C_* = organic *C* extracted from non-fumigated sediments; and *k_EC_* = 0.45 [38].

(2) $$MBN=\frac{E_{N}}{k_{EN}}=\frac{F_{N}-N_{N}}{k_{EN}}$$

where *F_N_* = total *N* extracted from fumigated sediments; *N* = total *N* extracted from non-fumigated sediments; and *k_EN_* = 0.54 [34].

(3) $$MBP=\frac{\frac{E_{P}}{k_{EP}}}{R_{P}}=\frac{\frac{F_{P}-N_{P}}{k_{EP}}}{1-\frac{F_{P}-N_{P}}{25}}$$

where *F_P_* = PO_4_^3^^−^-P extracted from fumigated sediment; *N_P_* = PO_4_^3^^−^-P extracted from non-fumigated sediment; and *k_EP_* = 0.40 [36].

Determination of sulfate content in the sediments (SO_4_^2−^) was undertaken as described by Gao et al. [39]. The SO_4_^2−^ content of the sediment was determined after the culture had been measured. All samples were run in triplicate.

Sediment phosphorus release rates (*V_P_*, mg m^−2^ d^−1^) were used to evaluate the amount of P transfer out of the sediment according to the following equation:

(4) $$V_{P}=\frac{\left(C_{after}-C_{before}\right)\times V}{S\times T}$$

where *C_after_* is the TP concentration in the overlying water in the sampling month after culturing (mg·L^−1^); *C_before_* is the TP concentration in the overlying water in the former month (mg·L^−1^); *V* is the volume of overlying water (mL); *S* is the area of the wetland sediment selected (m^2^); and *T* is the interval between two sampling dates (d). The sediment phosphorus release rates defined here provide a good indicator for assessing the P concentration changed by sediment phosphorus release, with high values suggesting a high capacity of P transfer from the sediment to overlying water. The sediment P release process and release rates are affected by various factors such as light time, temperature variation, pH value, oxygen concentration, biological activity, and *Microcystis* blooms etc. [32,40] while the chemical characteristics of the sediment were taken into consideration in this study. Therefore, all the chemical factors of the sediment that might affect the phosphorus release rates (*V_P_*) were selected. In addition, A, at the head of the indicators, means the chemical characteristics of the sediments after culturing. B, as well as A, represents the chemical characteristics of the sediments sampled originally and before culturing.

### 2.4. Statistical Analysis

The results shown in the tables are arithmetic average values of chemical indicators and measured on an oven-dry basis (about 24 h at 105 °C). All data except pH were ln-transformed before analysis to improve the normality of distribution. Correlation analysis was performed using OriginPro 9.1.0 (OriginLab Co., Northampton, UK). The significance of differences among samples was examined by one-way analysis of variance (ANOVA). ANOVA was performed using SAS 9.1 (SAS Institute Inc., Cary, NC, USA).

Correlation analysis was used to analyze the relationship between the sediment phosphorus release rates and the other driving factors. The major factors affecting phosphorus release rates were selected by the principal component analysis method. Based on the above analysis results, path analysis was used to investigate the relationship between the phosphorus release rates and the main driving factors, and the interrelation among the main driving factors. Principal component analysis is a statistical method to covert high-dimensional data into lower dimensional space [41]. Path analysis, a development of regression analysis, can be used to build a structured model through a hypothetical frame [42]. The determination coefficient is the relative determination degree of the reason for the results (DYX*i*X*j*); Y is the dependent variable (sediment phosphorus release rates in this research); X*i* and X*j* are the independent variables (any two factors in this experiment). If *i* = *j*, it means they are the same factor. All data were analyzed using SAS 9.1 and SPSS 19.0 (IBM SPSS Statistics, Armonk, NY, USA).

## 3. Results

### 3.1. Chemical Characters of Sediment in Types of Wetlands

The content of soil organic carbon (SOC) of the W–M was 59.11 ± 18.83 g·kg^−1^ and that of the W–L was 76.38 ± 48.91 g·kg^−1^. Both the sediments had lower SOC content compared with the W–R and W–A. Total nitrogen (TN) in the W–L was 5.73 ± 0.61 g·kg^−1^ comparedwith the W–M which had a TN concentration of 31.85 ± 7.86 g·kg^−1^. The W–R had a much lower total phosphorus (TP) content compared with the W–M. Based on Al-P, Fe-P, Ca-P and Oc-P concentrations in a NaHCO_3_ extract, the IP values were calculated by adding up these four concentrations for both sediments. The IP for the W–R was lower than that of W–M. The SO_4_^2−^ concentrations in the W–M was higher than that in the W–R, W–L and W–A, with concentrations of 5.39 ± 1.29 mg·kg^−1^, 2.83 ± 0.86 mg·kg^−1^ and 0.87 ± 0.11 mg·kg^−1^, respectively. The W–M had the higher nutrient concentrations, but the SOC concentrations were lower (Table 2).

### 3.2. Phosphorus Release Rates in Sediment Surface Water of Types of Wetlands

**Figure 3:**
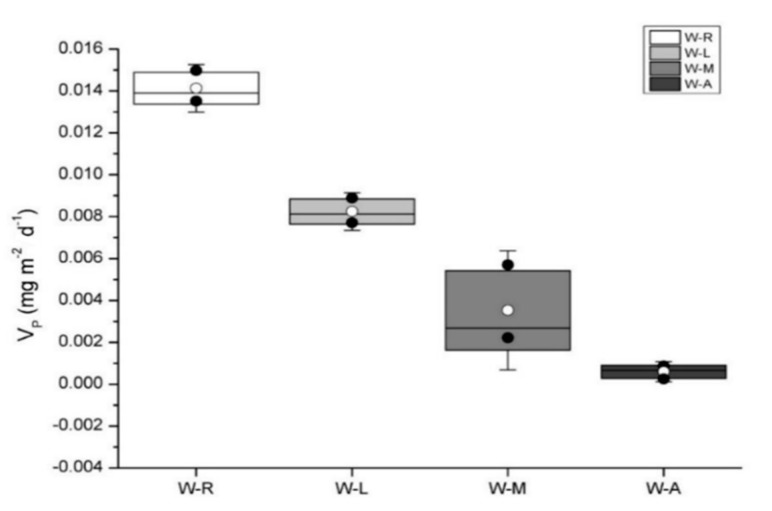
Variations of sediment phosphorus release rates in different wetlands.

A statistical summary of the *V_P_* of sediment and water samples collected from the types of wetland in the Hulunbeier steppe are presented in Figure 3. The highest average *V_P_* was observed in W–R followed by W–L, W–M and W–A and there was a significant difference between these four wetland sediments. This showed that different wetlands have different release rates; the highest one was W–R while the lowest was W–A.

### 3.3. Main Factors Affecting Phosphorus Release Rates

Correlation analysis Sediment phosphorus release rates were significantly and negatively correlated with the content of A-Fe-P, A-Ca-P, A-TP, B-Fe-P, B-MBC and B-TP (*r* = −0.499, −0.654, −0.538, −0.684, −0.560 and −0.546, respectively, *p* < 0.05; Table 3). Sediment phosphorus release rates decreased with the increase of the sediment content of A-Fe-P, A-Ca-P, A-TP, B-Fe-P, B-MBC and B-TP, but increased with the increase of the sediment B-SO4^2−^ and B-MBN content. The sediment phosphorus release rates are often lower when sediment total phosphorus is increased. The sediment A-Al-P content effects the sediment phosphorus release rates through sediment A-MBC and A-MBN concentrations.

Sediment phosphorus release rates were not correlated with A-MBP (*r* = 0.388), A-MBN (*r* = −0.069), A-MBC (*r* = −0.236) and B-MBP (*r* = 0.160). Sediment B-TP was significantly and positively correlated with A-Fe-P, A-Ca-P, A-TP, B-Ca-P and B-MBC (*r* = 0.612, 0.882, 0.998, 0.780 and 0.673 respectively, *p* < 0.01). Sediment A-TP was not correlated with sediment B-MBP (*r* = −0.097) and B-MBN (*r* = −0.285). Both sediments B-TP (0.361) and A-TP (*r* = 0.358) were not correlated with A-MBC. However, sediment B-MBC content was significantly and positively correlated with sediment A-TP (*r* = 0.677) and B-TP (*r* = 0.673) content. This indicates that the microbial biomass C content after culturing is influenced by the original TP content of the sediment and the TP content after the culturing of the sediment.

Principal component analysis The principal component analysis method, which can convert observations of possibly correlated variables into values of linearly uncorrelated variables, was used to determine the main factors of sediment phosphorus release rates (Table 4 and Table 5). The combined score of the former five principal components is 1.687, larger than 1 (Table 4). The cumulative variance contribution rate of the former four principal components was 92.057% (Table 4). Therefore, to determine the major factors influencing sediment phosphorus release rates, only the former four components were used for the next analytical step. The first principal component was analyzed as an important component and the other three components as supplements in order to determine the factors influencing sediment phosphorus release rates in the present study.

The component score coefficient matrix was used to select the driving factors in each principal component (Table 5). Sediment A-MBP and Sediment A-Ca-P content largely accounted for the first principal component in this analysis phase, with a characteristic vector of −0.182 and 0.165, respectively. This implies that sediment A-MBP and sediment A-Ca-P content were the main factors driving sediment phosphorus release rates. Sediment B-MBN (with a characteristic vector of 0.151) contributed less to sediment phosphorus release rates than sediment A-MBP. The sediment B-MBN content is highest in the second component (with a characteristic vector of −0.350). So, the sediment B-MBN content might have played a vital function in sediment phosphorus release rates. B-Oc-P is an important contributing factor impacting sediment phosphorus release rates and it is highest in the third component. Sediment A-Oc-P content and B-Fe-P content contributed significantly in the fourth component, but they contributed little in the former three components.

Overall, principal component analysis showed that sediment A-MBP, A-Ca-P, B-MBN, B-Oc-P, A-Oc-P and B-Fe-P content were more important factors affecting sediment phosphorus release rates, and should be considered when designing models for more robust simulation of sediment phosphorus release rates.

Path analysis Sediment B-SO_4_^2−^, B-MBN and A-MBP content were chosen as the major driving factors of sediment phosphorus release rates based on correlation analysis and principal component analysis. Path analysis was used to investigate the relationship between phosphorus release rates and these three factors, and to establish a model to describe the relative importance of direct and indirect effects of these three factors on sediment phosphorus release rates. The results of stepwise multiple regression analysis are shown in Table 6. Eventually, the established equation of the sediment phosphorus release rates was Y = −0.105 + 0.096X_1_ + 0.275X_2_ − 0.010X_3_ (*r* = 0.416, *p* < 0.01, *n* = 144), where Y is sediment phosphorus release rates; X_1_ is sediment B-SO_4_^2−^ content; X_2_ is sediment B-MBN; and X_3_ is sediment A-MBP content. Each coefficient in the equation was the direct path coefficient of each factor on phosphorus release rates. The indirect correlation coefficient between two factors was calculated by the direct path coefficient and the correlation coefficient between two factors (Table 7).

The sediment B-SO_4_^2−^, B-MBN and A-MBP content had a direct effect (the paired direct path coefficients are 0.884, 0.436, and −0.283, respectively) and indirect effect (the corresponding total indirect path coefficients are −0.036, 0.309, and 0.670, respectively) on sediment phosphorus release rates in the sediment–water interface (Table 7). This indicates that sediment B-SO_4_^2−^, B-MBN and A-MBP content could affect the microbial activity and decomposition of phosphorus fractions in the sediment and control sediment phosphorus release rates in the sediment–water interface, both directly and indirectly.

The determination coefficients influencing sediment phosphorus release rates factors were in the order of D_y_X_1_X_1_ (0.7815) > D_y_X_1_X_3_ (−0.3562) > D_y_X_1_X_2_ (0.2922) > D_y_X_2_X_2_ (0.1901) > the others (Table 8). This confirm that sediment B-SO_4_^2−^, B-MBN, A-MBP and the interactions between them were the main factors driving sediment phosphorus release rates.

## 4. Discussion

### 4.1. Phosphorus Release Rates

The *V_P_* of the sediment was arranged in the order of W–R > W–L > W–M > W-A. Under the same environmental conditions, the highest and lowest *V_P_* were shown in W–R and W–A, respectively (Figure 3). This may be related to the structure and composition of the sediments [43]. In general, the smaller the particle size, the better the adsorption capacity of phosphorus in the sediment and the less conducive to phosphorus release [44]. This is mainly due to the fact that the total specific surface of the coarse particles is much smaller than that of the fine particles when the weights of the particles are equal [45]. Sorption kinetics and isotherm curves of phosphate on different particle size fractions from the Wuli Lake and Gonghu Lake sediments have a similar trend, and the trend of different particle sizes fractions is: clay > fine sand > coarse sand > silt [46]. Considering the influence of the flow rate, it is difficult for the fine sand to settle in the sediment in W–R [47]. Therefore, the composition particle size of W–R is large and with a weakly capacity for phosphorus adsorption [48]. Consequently, the phosphorus release from the sediment of W–R occurs easily when exposed to environmental interference [49,50]. Simultaneously, the sources of water and pollutants in the W–R were multiple, and the composition of the sediment is complex [51,52,53]. The chemical reactions after temperature rises increase; the pH and redox state will change, which will lead to phosphorus release from sediment in the W–R [54]. That is the reason why the W–R has the highest *V_P_*. As for the W–A, its establishment time was short (completed in 2013); the pollutant accumulation was still lower. Meanwhile, because of the decrease of the water flow rate and disturbance, the increase of settlement is more favorable to the adsorption of phosphorus [55,56]. This might indicate that the W–A had a higher ability to maintain stability than the W–R when the environmental factors change, e.g., temperature changes. At the same time, in the three types of natural wetlands, although the phosphorus content of the W–M is very high, the initial resistance to temperature rises is high. It might have a strong buffering capacity to resist environmental change.

### 4.2. Factors Affecting Phosphorus Release Rate in Sediment–Water Interface

Based on the correlation analysis, the sediment phosphorus release rates and the main sediment factors were regressed. The results showed that the three driving factors were: sediment B-SO_4_^2−^, B-MBN and A-MBP content with a total impact of 98.20% on the sediment phosphorus release rates. It means these three factors could predict the change of the sediment phosphorus release rates well. The coefficient of *P_e_* can be used to analyze the influence of four independent variables on the sediment phosphorus release rates. This value is slightly larger, indicating that there are some factors not taken into account besides these four independent variables.

The results of path analysis showed that sediment B-SO_4_^2^^−^ content is the main determinant of the sediment phosphorus release rates, and it should be taken as the most important index to predict the sediment phosphorus release rates. Sulfate reduction to H_2_S and reoxidation by O_2_ to SO_4_^2^^−^ is a significant biogeochemical redox process with a high potential for acid generation (2H^+^ generated per H_2_S oxidized) and thus for CaCO_3_ dissolution [57]. Sulfates can oxidize manganese and iron complexes [58]. The change of redox conditions led to the phosphorus release from the sediments.

Soil microbial biomass phosphorus refers to the phosphorus contained in all living microorganisms in the soils, and is the main component of nucleic acid and phosphorus content in the soil. It is very small, usually accounting for 1.4% to 4.7% of the dry weight of microorganisms [37,59,60]. However, because the turnover rate of soil microbial biomass phosphorus is rapid, it is an important source of effective phosphorus in plants, and is of great significance for regulating the plant availability of phosphorus in soil and the biogeochemical cycling of phosphorus [61,62,63]. Simultaneously, soil microbial biomass phosphorus is sensitive to environmental changes, and an accurate determination of microbial biomass phosphorus content in soil contributes to a better understanding of the phosphorus fixation and turnover caused by the environmental change (climate, soil type, topography change) and human activities (fertilizer, pesticides, crop cover, tillage); and so the effectiveness of soil fertility and soil nutrients have important significance [64,65]. In summer, microbial activity is enhanced, which will consume large amounts of nutrients; when the energy supply is insufficient, microbial activity weakens and the biomass decreases, so the sediment has the potential for biological phosphorus release.

Soil microbial biomass N is not only an executor of microbial mineralization and fixation of soil nitrogen, but also an active pool for providing nutrients to plants [66,67] and regulating the soil nitrogen supply directly. Therefore, the activity of soil microbial biomass nitrogen and its growth and decline are considered to be essential contents of soil nitrogen internal circulation [68]. It was found that MBN had an effect on the release rate of phosphorus in sediments, which may be due to microbial biomass N:P and the ecological stoichiometry that affected the release of phosphorus from the sediments [69].

Therefore, it is necessary to consider sediment B-SO_4_^2^^−^, B-MBN and A-MBP content in order to predict and control the sediment phosphorus release rates while maintaining the other physical and chemical properties of the sediment.

## 5. Conclusions

The results showed that the *V_P_* of different wetland sediments were different and changed in the order of W–R > W–L > W–M > W–A.

Sediment phosphorus release rates in the sediment–water interface were closely and significantly correlated with the content of A-Fe-P, A-Ca-P, A-TP, B-Fe-P, B-MBC and B-TP. The main driving factors that influenced sediment phosphorus release rates in the sediment–water interface were sediment B-SO_4_^2^^−^, B-MBN and A-MBP content, and the standard multiple regression equation for sediment phosphorus release rates in the sediment–water interface and main affecting factor was Y = −0.105 + 0.096X_1_ + 0.275X_2_ − 0.010X_3_ (*r* = 0.416, *p* < 0.01, *n* = 144).

Therefore, these results suggest that sediment chemical properties and microbial activities, likely play an important role in phosphorus release rates in the sediment–water interface. Future research will take sediment chemical properties, microbial activities, vegetation factors and other environmental factors into account, in order to analyze the impact of all external and internal factors on phosphorus release in the steppe wetlands. It hopes to provide effective scientific management and control methods for relevant environmental protection departments.

## Figures and Tables

**Table 1:** Site details of the six wetlands selected in the study.

Name of Sampling Place	Abbreviation	Latitude and Longitude	Statues of the Wetland	Average Water Depth (m)	Wetland Types
HuiheMuqiao	R1	119°02′21.89″ E	The surroundings are grazing steppe.	1.0–2.5	River wetland (W–R)
		48°27′14.42″ N			
Interchange of Huihe and Yimin River	R2	119°44′09.23″ E	The surroundings are grazing steppe.	1.0–2.5	
		49°01′50.27″ N			
Hulun Lake	L1	117°27′31.25″ E	The surroundings are grazing steppe.	4.0–5.5	Lake wetland (W–L)
		49°11′23.88″ N			
Swan Lake	L2	119°04′21.99″ E	The surroundings are grazing steppe.	2.0–3.0	
		48°28′43.62″ N			
HuiheXiboqiao	M	119°13′50.42″ E	The surroundings are grazing steppe.	1.0–2.5	Grassy Marsh wetland (W–M)
		48°49′13.25″ N			
Midstream of Yimin River	A	120°01′44.28″ E	Water sources protection area.	0.–2.5	Reservoir wetland (W–A)
		48°16′59.52″ N			

**Table 2:** Chemical characteristics of the four types of wetlands selected in the study.

Wetland Types	SOC g·kg^−1^	TN g·kg^−1^	TP mg·kg^−1^	IP mg·kg^−1^	SO_4_^2−^ mg·kg^−1^
W–R	145.78 ± 2.01 a	7.16 ± 0.39 bc	507.53 ± 13.79 d	407.08 ± 9.60 d	5.39 ± 1.29 b
W–L	76.38 ± 48.91 ab	5.73 ± 0.61 c	747.13 ± 32.27 c	584.36 ± 13.29 c	2.83 ± 0.86 b
W–M	59.11 ± 18.83 b	31.85 ± 7.86 a	1057.56 ± 51.81 a	766.99 ± 8.75 a	23.83 ± 5.95 a
W–A	146.08 ± 61.36 a	15.10 ± 2.96 b	894.65 ± 15.54 b	629.13 ± 9.58 b	0.87 ± 0.11 b

Note: Average values ± SD (standard deviation) of chemical characteristics of types of wetlands. Lower case letters, such as a, b, c and d, indicate differences between different factors. The same letters mean the difference is not significant, and different letters indicate significant differences between two wetland types at the 0.05 level.

**Table 3:** The correlation coefficient of sediment P release rates (*V_P_*) and other factors (unit: mg/kg).

Item	*Vp* (mg/m^−2^/d^−1^)	A-Al-P	A-Fe-P	A-Ca-P	A-Oc-P	A-MBP	A-MBC	A-MBN	A-TP	B-SO_4_^2−^	B-Al-P	B-Fe-P	B-Ca-P	B-Oc-P	B-MBP	B-MBC	B-MBN	B-TP
*Vp* (mg/m^−2^/d^−1^)	1.000	0.015	−0.499 *	−0.654 **	0.336	0.388	−0.236	−0.069	−0.538 *	0.848 **	−0.230	−0.684 **	−0.362	−0.191	0.160	−0.560 *	0.744 **	−0.546 *
A-Al-P		1.000	−0.349	0.226	−0.225	−0.174	−0.484 *	−0.536 *	0.235	−0.241	0.844 **	0.595 **	−0.296	0.653 **	−0.512 *	−0.295	0.399	0.231
A-Fe-P			1.000	0.313	0.445	−0.225	0.870 **	0.683 **	0.614 **	−0.267	−0.511 *	0.042	0.797 **	−0.177	0.623 **	0.678 **	−0.648 **	0.612 **
A-Ca-P				1.000	0.051	−0.859 **	0.050	−0.166	0.878 **	−0.885 **	0.322	0.353	0.593 **	0.460	−0.462	0.619 **	−0.276	0.882 **
A-Oc-P					1.000	−0.149	0.382	0.557 *	0.361	0.366	−0.506 *	−0.608 **	0.464	0.133	0.590 **	0.089	−0.036	0.360
A-MBP						1.000	−0.047	0.158	−0.787 **	0.712 **	−0.214	−0.150	−0.572 *	−0.363	0.414	−0.520 *	0.093	−0.790 **
A-MBC							1.000	0.573 *	0.358	−0.042	−0.678 **	−0.166	0.721 **	−0.469 *	0.632 **	0.635 **	−0.408	0.361
A-MBN								1.000	0.093	0.272	−0.677 **	−0.315	0.412	−0.286	0.834 **	0.160	−0.513 *	0.106
A-TP									1.000	−0.701 **	0.111	0.262	0.779 **	0.398	−0.097	0.677 **	−0.285	0.998 **
B-SO_4_^2−^										1.000	−0.439	−0.646 **	−0.425	−0.315	0.550 *	−0.607 **	0.379	−0.704 **
B-Al-P											1.000	0.749 **	−0.492 *	0.750 **	−0.739 **	−0.367	0.153	0.111
B-Fe-P												1.000	−0.189	0.469 *	−0.393	0.006	−0.348	0.262
B-Ca-P													1.000	−0.227	0.251	0.890 **	−0.267	0.780 **
B-Oc-P														1.000	−0.370	−0.329	−0.102	0.397
B-MBP															1.000	0.025	−0.373	−0.100
B-MBC																1.000	−0.292	0.673 **
B-MBN																	1.000	−0.287
B-TP																		1.000

Note: * Correlation is significant at the 0.05 level-2-tailed; ** Correlation is significant at the 0.01 level-2-tailed.

**Table 4:** Total variance explained.

Component	Initial Eigenvalues			Extraction Sums of Squared Loadings		
	Total	Variance (%)	Cumulative (%)	Total	Variance (%)	Cumulative (%)
1	6.432	37.835	37.835	6.432	37.835	37.835
2	5.787	34.04	71.875	5.787	34.04	71.875
3	1.744	10.259	82.134	1.744	10.259	82.134
4	1.687	9.924	92.057	1.687	9.924	92.057
5	0.787	4.63	96.687			
6	0.254	1.497	98.184			
7	0.177	1.039	99.223			
8	0.081	0.479	99.702			
9	0.021	0.126	99.829			
10	0.014	0.083	99.912			
11	0.009	0.053	99.965			
12	0.004	0.022	99.987			
13	0.001	0.007	99.993			
14	0.001	0.005	99.998			
15	0	0.001	100			
16	0	0	100			
17	0	0	100			

**Table 5:** Component score coefficient matrix of factors affecting phosphorus release rates of the sediment.

Sediment Factor	Component			
	1	2	3	4
A-Al-P mg/kg	0.018	−0.020	0.233	0.066
A-Fe-P mg/kg	0.036	0.227	0.014	−0.045
A-Ca-P mg/kg	0.165	−0.052	0.019	−0.010
A-Oc-P mg/kg	0.058	0.039	0.179	0.420
A-MBP mg/kg	−0.182	0.131	0.008	−0.088
A-MBC mg/g	0.032	0.127	−0.114	−0.047
A-MBN mg/g	−0.054	0.252	0.080	0.067
A-TP mg/kg	0.158	0.024	0.089	0.090
B-SO_4_^2−^ mg/kg	−0.113	−0.001	0.056	0.234
B-Al-P mg/kg	−0.009	0.008	0.201	−0.089
B-Fe-P mg/kg	−0.047	0.204	0.138	−0.322
B-Ca-P mg/kg	0.158	−0.037	−0.126	0.073
B-Oc-P mg/kg	0.011	0.111	0.375	0.140
B-MBP mg/kg	−0.090	0.249	0.082	0.094
B-MBC mg/g	0.151	−0.073	−0.242	−0.108
B-MBN mg/g	0.064	−0.350	−0.090	0.245
B-TP mg/kg	0.158	0.025	0.089	0.089

**Table 6:** The standard multiple regression coefficient of main factors affecting sediment phosphorus release rates.

Model	Unstandardized Coefficients			Standardized Coefficients		
	Item	B	Std. Error	Beta	t	Sig.
1	(Constant)	−0.087	0.027		−3.25	0.005
	B-SO_4_^2−^mg/kg	0.092	0.014	0.848	6.396	0
2	(Constant)	−0.1	0.014		−7.009	0
	B-SO_4_^2−^mg/kg	0.072	0.008	0.661	8.807	0
	B-MBN mg/g	0.312	0.047	0.494	6.585	0
3	(Constant)	−0.105	0.01		−10.017	0
	B-SO_4_^2−^mg/kg	0.096	0.009	0.884	10.973	0
	B-MBN mg/g	0.275	0.036	0.436	7.669	0
	A-MBP mg/kg	−0.01	0.003	−0.283	−3.776	0.002

**Table 7:** Path coefficient of each factor on sediment phosphorus release rates.

Sediment Factor	Correlation Coefficient	Direct Path Coefficient	Indirect Path Coefficient			
			X_1_ (B-SO_4_^2−^)	X_2_ (B-MBN)	X_3_ (A-MBP)	Total
X_1_ (B-SO_4_^2^^−^)	0.848	0.884		0.165	−0.202	−0.036
X_2_ (B-MBN)	0.744	0.436	0.335		−0.026	0.309
X_3_ (A-MBP)	0.388	−0.283	0.629	0.041		0.670

**Table 8:** The determination coefficient of each factor affects the sediment phosphorus release rates.

Sediment Factor	X_1_ (B-SO_4_^2−^)	X_2_ (B-MBN)	X_3_ (A-MBP)
X_1_ (B-SO_4_^2−)^	0.7815	0.2922	−0.3562
X_2_ (B-MBN)		0.1901	−0.0230
X_3_ (A-MBP)			0.0801
